# Development of a lung immune prognostic index-based nomogram model for predicting overall survival and immune-related adverse events in non-small cell lung cancer patients treated with sintilimab

**DOI:** 10.3389/fimmu.2025.1569689

**Published:** 2025-05-08

**Authors:** Jian Xu, Tingting Peng, Kaikai Fan, Yuxiao Dou, Lingti Kong, Ran Sang

**Affiliations:** ^1^ Department of Pharmacy, The First Affiliated Hospital of Bengbu Medical University, Bengbu, China; ^2^ Department of Spinal Surgery, The First Affiliated Hospital of Bengbu Medical University, Bengbu, China; ^3^ Department of Pharmacy, Suzhou Municipal Hospital, Suzhou, China; ^4^ Department of Pharmacy, Hefei Eighth People’s Hospital, Hefei, China

**Keywords:** nomograms, non-small cell lung cancer, immunotherapy, inflammation markers, predicting, sintilimab

## Abstract

**Background:**

Sintilimab, a programmed cell death protein-1 (PD-1) inhibitor, has shown efficacy in non-small cell lung cancer (NSCLC), though response heterogeneity persists. Previous studies suggest that the Lung Immune Prognostic Index (LIPI) may predict prognosis and immune-related adverse events (irAEs) in immunotherapy. This study aimed to develop and validate LIPI-based nomograms for predicting overall survival (OS) and irAEs in NSCLC patients treated with sintilimab.

**Methods:**

Multicenter data stratified 356 patients into training, internal validation, and external validation cohorts. Propensity score matching (PSM) balanced baseline characteristics. Multivariable Cox regression identified OS and irAEs predictors, and nomograms were constructed using significant variables. Model performance was evaluated via concordance index (C-index), time-dependent receiver operating characteristic (ROC) curves, calibration plots, and decision curve analysis (DCA). Kaplan-Meier analysis assessed risk stratification.

**Results:**

Independent prognostic factors for OS include clinical stage, treatment lines, LIPI scores and albumin level. Among them, stage IV (hazard ratio [HR]=1.725, 95% confidence interval [CI] 1.529-1.902), treatment line ≥2 (HR=1.302, 95%CI: 1.125-1.569), LIPI intermediate (HR=1.736, 95%CI: 1.586-1.925), LIPI poor (HR=1.568, 95% CI: 1.361-1.637) and albumin level≥35 (HR=1.802, 95%CI: 1.698-2.023) were risk factors for OS. The OS prediction model demonstrated excellent discrimination across all cohorts, with time-dependent AUCs maintaining 0.770-0.850 for 1–2 year predictions. Consistent calibration was observed (C-index: training=0.778, internal validation=0.793, external validation=0.790). For irAEs prediction, significant predictors included age, sex, Eastern Cooperative Oncology Group performance status (ECOG PS), and LIPI scores. Similarly, the irAEs model showed robust performance (AUCs 0.754-0.835 for 1–2 year predictions; C-index: training=0.805, internal validation=0.825, external validation=0.775). Both nomograms significantly outperformed single-variable predictions in Kaplan-Meier analyses. DCA confirmed superior net clinical benefit.

**Conclusion:**

LIPI-based nomograms effectively predicted OS and irAEs in sintilimab-treated NSCLC patients, offering valuable tools for personalized treatment and clinical decision-making.

## 1 Introduction

Non-small cell lung cancer (NSCLC) remains the leading cause of cancer-related deaths worldwide, accounting for 85% of lung cancer cases and approximately 1.8 million deaths annually (1). Current NSCLC treatments include chemotherapy, targeted therapy, and immunotherapy, with the latter representing a paradigm shift (2). Sintilimab, a programmed death-1 (PD-1) inhibitor developed in China, has become a cornerstone therapy for NSCLC, demonstrating significant survival benefits in first- and second-line treatments (3, 4). However, response heterogeneity and immune-related adverse events (irAEs) pose challenges to its broader clinical adoption (5).

While biomarkers like programmed death-ligand 1 (PD-L1) expression and tumor mutation burden (TMB) have been explored for predicting anti-PD-1 therapy efficacy, their clinical utility remains inconsistent (6). The Lung Immune Prognostic Index (LIPI), a non-invasive inflammatory biomarker derived from routine blood tests, has shown prognostic value in multiple cancers (7, 8). Prior studies (9–11) further suggest associations between LIPI and survival or irAEs in sintilimab-treated NSCLC patients.

In this study, we established a nomogram model incorporating the LIPI to predict 1- and 2-year OS and irAEs in sintilimab-treated NSCLC patients. This work advances the field by addressing the unmet need for sintilimab-specific predictive tools, bridging a critical gap in the literature regarding LIPI and nomogram applications in NSCLC immunotherapy. The clinical imperative for sintilimab-oriented predictive models is underscored by their potential to enable clinicians to stratify risks and optimize therapeutic strategies during immunotherapy. Beyond immediate decision-making, this model may reshape clinical practice paradigms for NSCLC, enhancing survival benefits while mitigating irAEs through proactive management.

## 2 Materials and methods

### 2.1 Study design and patient selection

This retrospective, multicenter study evaluated data from patients with advanced or metastatic non-small cell lung cancer (NSCLC) who received sintilimab therapy at three tertiary centers: the First Affiliated Hospital of Bengbu Medical University, Suzhou Municipal Hospital, and Hefei Eighth People’s Hospital, between January 1, 2019, and January 1, 2023. Patients meeting the following inclusion criteria were enrolled: (1) diagnosis of advanced or metastatic NSCLC confirmed pathologically, in accordance with the Chinese Society of Clinical Oncology (CSCO) Guidelines for Primary Lung Cancer (12, 13); (2) American Joint Committee on Cancer (AJCC) stage IIIB-IV (14); (3) deemed unsuitable for surgical treatment; (4) received sintilimab as first- or second-line therapy; (5) completed at least two cycles of sintilimab; and (6) availability of baseline imaging, blood tests, and clinical data. Patients were excluded if they met any of the following criteria: (1) presence of a concurrent second primary tumor or multiple primary tumors; (2) lack of complete blood count or biochemical test results within 14 days prior to treatment initiation; (3) use of anticoagulant or antiplatelet medications; or (4) follow-up duration of less than one month.

Based on these criteria, 1,197 patients were included: 806 from the First Affiliated Hospital of Bengbu Medical University, 181 from Hefei Eighth People’s Hospital, and 210 from Suzhou Municipal Hospital. Of the Bengbu cohort, 538 patients were randomly assigned to the training set for prognostic model development, while 268 patients formed the internal validation set. An additional 391 patients from Hefei Eighth People’s Hospital and Suzhou Municipal Hospital constituted the external validation set. The institutional review boards of all three participating centers approved this retrospective observational study, and the requirement for informed consent was waived due to its retrospective design.

### 2.2 Data collection

A comprehensive set of variables was collected for analysis, including age, sex, Eastern Cooperative Oncology Group performance status (ECOG-PS), pathological subtype, clinical stage, history of radiotherapy, treatment lines, lactate dehydrogenase (LDH) levels within 14 days prior to sintilimab treatment, neutrophil count (NEUT), white blood cell count, hemoglobin (Hb) level, albumin (ALB) level, carbohydrate antigen 199 (CA199), epidermal growth factor receptor (EGFR) status, anaplastic lymphoma kinase (ALK) status, programmed death-ligand 1 tumor proportion score (PD-L1 TPS), overall survival (OS), and immune-related adverse events (irAEs) (15).

The LIPI was calculated using two biomarkers: the derived neutrophil-to-lymphocyte ratio (dNLR) and LDH levels. The LIPI score was determined as follows (16): (1) dNLR > 3 was assigned 1 point, while dNLR ≤ 3 received 0 points; (2) LDH > 245 IU/L was assigned 1 point, while LDH ≤ 245 IU/L received 0 points; (3) Total scores were categorized into three groups: 0 (good prognosis), 1 (intermediate prognosis), and 2 (poor prognosis).

### 2.3 Outcome definition and follow-up

In this study, OS and the occurrence of irAEs were primary outcomes. OS was defined as the time interval from the first administration of sintilimab immunotherapy to death from any cause. Patients who were alive at the last follow-up were censored. OS calculation began at the initiation of treatment. irAEs were defined as adverse events occurring during or after sintilimab treatment that met the following criteria: (1) Temporally associated with sintilimab treatment (occurring within a clinically plausible timeframe); (2) Clinically consistent with known sintilimab-related irAEs; (3) Not attributable to alternative causes (e.g., infection, disease progression); and (4) Responsive to immunosuppressive therapy. Events were graded according to the Common Terminology Criteria for Adverse Events (CTCAE) v5.0. Diagnosis of irAEs required a comprehensive clinical, laboratory, and imaging evaluation.

This retrospective study involved a review of medical records, inpatient electronic medical records, and laboratory test results from the First Affiliated Hospital of Bengbu Medical University, Hefei Eighth People’s Hospital, and Suzhou Municipal Hospital. Follow-up data were obtained through questionnaires and telephone interviews to minimize data loss and bias. Collected follow-up information included the patient’s clinical condition, tumor treatment status, occurrence of adverse reactions, and survival status. If there was no disease progression, death, or irAEs by the last follow-up date, or if the patient was lost to follow-up, the data were treated as censored.

### 2.4 PSM

For ordinal data, nonparametric tests were applied. When baseline characteristics showed imbalance (*P* < 0.05) across the training, internal validation, and external validation sets, propensity scores were calculated using logistic regression, followed by propensity score matching (PSM) (17). PSM was conducted using R software (version 4.1.12; Institute for Statistics and Mathematics, Vienna, Austria) with the “pm3” package. A 2:1:1 matching ratio was applied using the nearest neighbor algorithm without replacement, with a caliper width of 0.1

### 2.5 Model development and evaluation

Univariate Cox regression analysis was initially performed to identify statistically significant variables, which were then included in a multivariate Cox regression model using forward stepwise selection to determine independent predictors for OS and irAEs. Based on the multivariate Cox regression results from the training cohort, nomogram models were developed to predict OS and irAEs. Time-dependent receiver operator characteristics (ROC) curves and Concordance Index (C-index) were used to evaluate the discrimination ability of the nomogram (18). Calibration curves were generated to assess the calibration ability of the nomogram by observing the consistency between the actual- and predicted OS/irAEs. Kaplan-Meier survival curves along with log-rank test were adopted to evaluate the risk stratification ability of the nomogram (19). Ultimately, decision curve analysis (DCA) was also performed to evaluate the clinical utility and net benefit of the nomograms via “dcurves” package.

### 2.6 Statistical analysis

Continuous variables were analyzed using t-tests or nonparametric tests, while categorical variables were assessed using Fisher’s exact test. Inter-cohort comparisons (training, internal validation, and external validation) were conducted using Mann-Whitney U tests for continuous variables, while Fisher’s exact tests were employed for categorical variables, depending on expected frequencies. Prognostic factors were initially identified through univariate Cox regression analysis, with significant variables (*p*<0.1) subsequently incorporated into multivariate Cox proportional hazards models (SPSS version 23.0; IBM Corp., Armonk, NY, USA). The predictive accuracy of nomograms was assessed through multiple approaches: C-index for overall discriminative ability; ROC analysis with corresponding AUC values at clinically relevant timepoints; and Kaplan-Meier survival stratification. Between-group survival differences evaluated by log-rank testing. All statistical tests were two-sided, with a threshold of *p*<0.05 establishing statistical significance. Analytical procedures were performed using SPSS (version 23.0) and R statistical software (version 4.1.12; Institute for Statistics and Mathematics, Vienna, Austria).

## 3 Results

### 3.1 Clinical characteristics of patients

**Figure 1:**
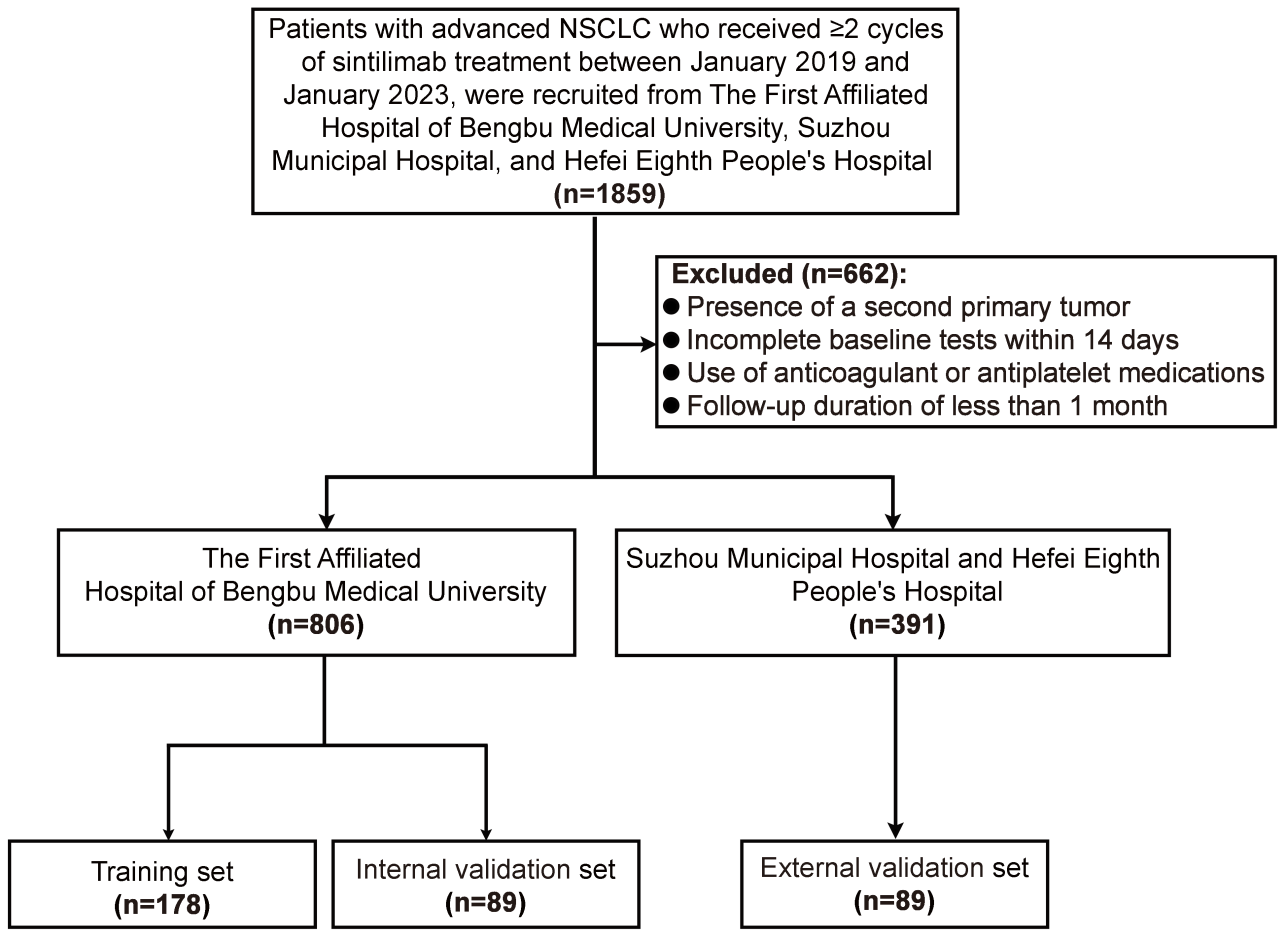
Flowchart illustrating the selection process for the retrospective study.

To ensure balanced baseline characteristics among the training set (n=538), internal validation set (n=268), and external validation set (n=391), PSM was performed at a 2:1:1 ratio. Post-matching, the datasets comprised a training set (n=178), an internal validation set (n=89), and an external validation set (n=89) (**Figure 1**). In the matched training set, 133 patients (74.72%) were male and 45 (25.28%) were female. The majority of patients (57.30%) were aged ≥60 years. Non-smokers accounted for 55.06% of the cohort, compared to 44.94% who were smokers. Most patients presented with stage IV disease (62.36%). Before PSM, significant differences were observed across the sets for variables including age, sex, pathological type, clinical stage, LIPI score, Hb, ALB, and CA199 levels (all *P* < 0.05). However, after applying PSM, all clinical characteristics were well-balanced across the sets (**Table 1**).

**Table 1:** Clinical characteristics of patients in the training and validation sets before and after propensity score matching (PSM).

Clinical characteristics	Before matching (n, %)			After matching (n, %)		
	Training set (n=538)	Internal validation set (n=268)	External validation set (n=391)	Training set (n=178)	Internal validation set (n=89)	External validation set (n=89)
Age (years)						
≥ 60	323 (60.04%)	108 (40.30%) ^*^	201 (51.41%)*	102 (57.30%)	50 (56.18%)	53 (59.55%)
<60	205 (38.10%)	160 (59.70%)	190 (48.59%)	76 (42.70%)	39 (43.82%)	36 (40.45%)
Sex						
Male	430 (79.93%)	181 (67.54%)*	254 (64.96%)*	133 (74.72%)	69 (77.53%)	62 (69.66%)
Female	108 (20.07%)	87 (32.46%)	137 (35.12%)	45 (25.28%)	20 (22.47%)	27 (30.34%)
ECOG-PS						
0~1	247 (45.91%)	136 (50.75%)	135 (34.52%)*	81 (45.51%)	38 (42.70%)	41 (46.07%)
≥2	291 (54.09%)	132 (49.25%)	256 (65.47%)	97 (54.49%)	51 (57.30%)	48 (53.93%)
Smoking						
Yes	241 (44.80%)	124 (46.27%)	215 (54.98%)*	80 (44.94%)	42 (47.19%)	39 (43.82%)
No	297 (55.20%)	144 (53.73%)	176 (45.01%)	98 (55.06%)	47 (52.81%)	50 (56.18%)
Pathological type						
Squamous cell carcinoma	192 (35.69%)	112 (41.79%)*	129 (32.99%)	65 (36.52%)	32 (35.96%)	34 (38.2%)
Non-squamous cell carcinoma	283 (52.60%)	107 (39.93%)	198 (50.63%)	74 (41.57%)	38 (42.70%)	37 (41.57%)
Others	63 (11.71%)	49 (18.28%)	64 (16.36%)	39 (21.91%)	19 (21.35%)	18 (20.22%)
Clinical staging						
Stage IIIB~IIIC	182 (33.83%)	125 (46.64%)*	140 (35.81%)	67 (37.64%)	33 (37.08%)	36 (40.45%)
Stage IV	356 (66.17%)	143 (53.35%)	251 (64.19%)	111 (62.36%)	56 (62.92%)	53 (59.55%)
History of radiotherapy						
Yes	53 (9.85%)	23 (8.58%)	54 (14.06%)*	16 (8.99%)	7 (7.87%)	9 (10.11%)
No	485 (90.15%)	245 (91.42%)	337 (86.19%)	162 (91.01%)	82 (92.13%)	80 (89.89%)
Treatment lines						
1	259 (48.14%)	129 (48.13%)	156 (39.89%)*	85 (47.75%)	44 (49.44%)	46 (51.69%)
≥2	279 (51.86%)	139 (51.87%)	235 (60.10%)	93 (52.25%)	45 (50.56%)	43 (48.31%)
LIPI						
Good	328 (60.97%)	108 (40.30%)*	198 (50.64%)*	97 (54.49%)	48 (53.93%)	45 (50.56%)
Intermediate	161 (29.93%)	142 (52.99%)	149 (38.11%)	62 (34.83%)	30 (33.71%)	29 (32.58%)
Poor	49 (9.11%)	18 (6.72%)	44 (11.25%)	19 (10.67%)	11 (12.36%)	15 (16.85%)
Tumor stage						
0-2	336 (62.45%)	116 (43.28%)*	231 (59.08%)	101 (56.74%)	57 (64.04%)	52 (58.43%)
3-4	202 (37.55%)	152 (56.72%)	160 (40.92%)	77 (43.26%)	32 (35.96%)	37 (41.57%)
EGFR/ALK						
Negative	306 (56.88%)	191 (71.27%)*	242 (61.89%)	116 (65.17%)	59 (66.29%)	55 (61.79%)
Unknown	232 (43.12%)	77 (28.73%)*	149 (38.11%)	62 (34.83%)	30 (33.71%)	34 (38.20%)
PD-L1 TPS						
< 1%	145 (26.95%)	59 (22.02%)	71 (18.15%)*	38 (21.34%)	17 (19.10%)	21 (23.59%)
≥ 1%	59 (10.97%)	22 (8.21%)	36 (9.21%)	21 (11.80%)	10 (11.24%)	9 (10.11%)
Unknown	334 (62.08%)	187 (69.77%)	284 (72.63%)*	119 (66.85%)	62 (69.66%)	59 (66.29%)
Hemoglobin						
<110	208 (38.66%)	136 (50.75%)*	144 (36.82%)	92 (51.69%)	43 (48.31%)	38 (42.7%)
≥110	330 (61.34%)	132 (49.25%)	247 (63.17%)	86 (48.31%)	46 (51.69%)	51 (57.3%)
Albumin						
<35	125 (23.23%)	85 (31.72%)*	105 (26.85%)	36 (20.22%)	19 (21.35%)	22 (24.72%)
≥35	413 (76.77%)	183 (68.28%)	286 (73.14%)	142 (79.78%)	70 (78.65%)	67 (75.28%)
CA199						
<37	348 (64.68%)	120 (44.78%)*	203 (51.92%)*	92 (51.69%)	46 (51.69%)	42 (47.19%)
≥37	190 (35.32%)	148 (55.22%)	188 (48.08%)	86 (48.31%)	43 (48.31%)	47 (52.81%)

### 3.2 Independent prognostic factors of OS

**Figure 2:**
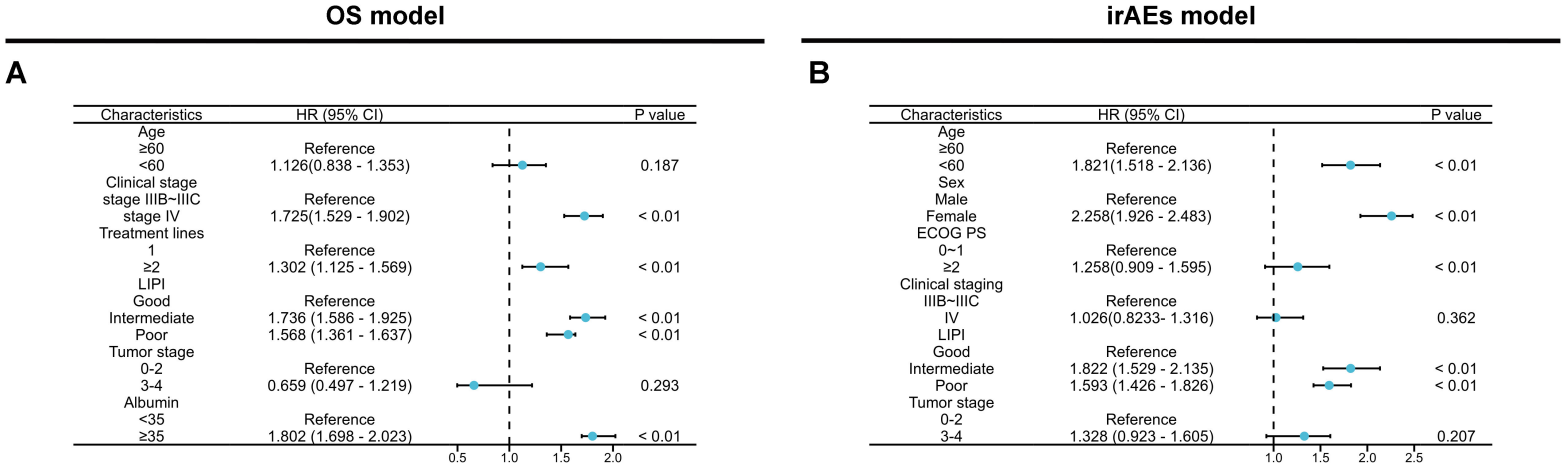
Multivariate Cox regression analysis of OS **(A)** and irAEs **(B)** across all features in the training set.

In the current study, as of the last follow-up, survival analysis revealed a median OS of 10.12 months (95% CI: 9.67-10.57, **Supplementary Figure S1A**) for the training cohort. The median OS for the internal validation cohort and external validation cohort were 14.27 months (95% CI: 13.05-16.11, **Supplementary Figure S1B**) and 17.05 months (95% CI:16.12-19.06, **Supplementary Figure S1C**), respectively. The correlation between 13 clinicopathological variables and OS was analyzed. Univariate analysis revealed six variables significantly associated with OS, including age, clinical stage, treatment line, LIPI group, ALB level, and T stage (**Supplementary Table S1**). These six variables were subsequently included in multivariate Cox regression analysis, which identified four independent risk factors for OS: stage IV (HR=1.725, 95% CI: 1.529–1.902, *p* < 0.01), treatment line ≥2 (HR=1.302, 95% CI: 1.125–1.569, *p* < 0.01), LIPI intermediate (HR=1.736, 95%CI: 1.586-0.925, *p* < 0.01) and LIPI poor(HR=1.568, 95% CI: 1.361–1.637, *p* < 0.01), and ALB value ≥35 (HR=1.802, 95% CI: 1.698–2.023, *p* < 0.01) (**Figure 2A**).

### 3.3 Independent prognostic factors of irAEs

**Figure 3:**
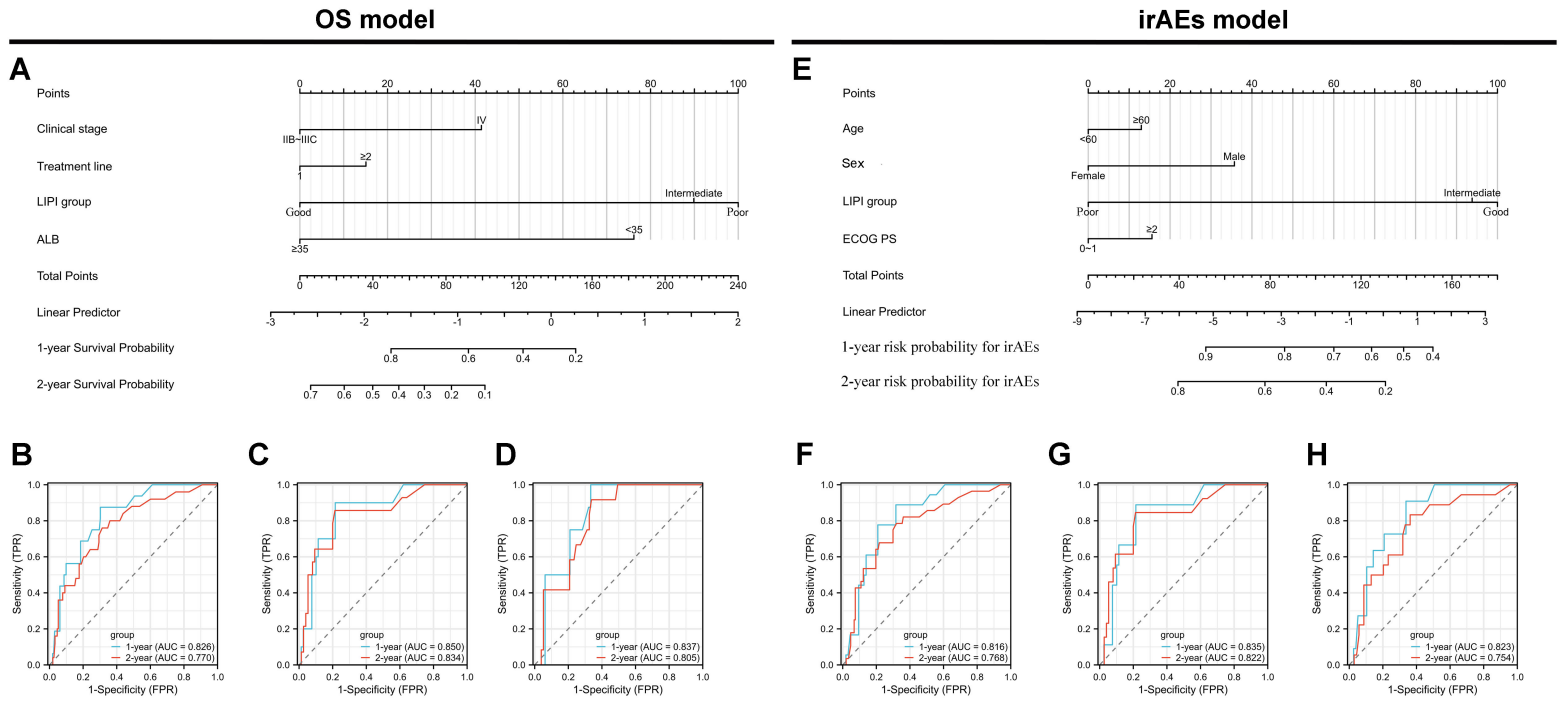
Development and validation of nomogram models for overall survival (OS) and immune-related adverse events (irAEs) in advanced non-small cell lung cancer (NSCLC) patients treated with Sintilimab. **(A)** Nomogram model for predicting the 1-year and 2-year OS of patients with advanced NSCLC. **(B–D)** Receiver operating characteristic (ROC) curves for 1-year and 2-year OS predictions in the training set **(B)**, internal validation set **(C)**, and external validation set **(D)**. **(E)** Nomogram model for predicting the 1-year and 2-year probabilities of irAEs in patients with advanced NSCLC. **(F–H)** ROC curves for 1-year and 2-year irAEs predictions in the training set **(F)**, internal validation set **(G)**, and external validation set **(H)**.

As of the last follow-up, the median time of irAEs occurrence in the training cohort was 11.86 months (95% CI: 10.43-12.57, **Supplementary Figure S1D**), while the median time of irAEs occurrence in the internal validation cohort and external validation cohort were 11.67 months (95% CI: 8.16-12.67, **Supplementary Figure S1E**) and 11.81 months (95% CI: 7.86-13.11, **Supplementary Figure S1F**), respectively. Univariate analysis of irAEs, revealed that age <60(HR=1.688, 95% CI: 1.236–2.132, *p* < 0.01), female (HR=1.625, 95% CI: 1.321–2.086, *p <*0.01), ECOG score ≥2(HR=3.358, 95% CI: 2.765–4.532,: *p* < 0.01), stage IV (HR=1.982, 95% CI: 1.428–2.392, *p <*0.01), LIPI intermediate (HR=1.957, 95% CI: 1.657–2.361, *p <*0.01) and LIPI poor(HR=1.757, 95% CI: 1.526–1.933, *p* < 0.01), and T stage 3-4(HR=2.657, 95% CI: 2.328–2.863, *p <*0.01) were significantly related to irAEs (*p* < 0.05) (**Supplementary Table S2**). Cox multivariable analysis revealed that age <60 (HR=1.821, 95% CI: 1.518–2.136, *p* < 0.01), female(HR=2.258, 95% CI: 1.926–2.483, *p <*0.01), ECOG score ≥2(HR=1.258, 95% CI: 0.909–1.595, *p* < 0.01), and LIPI intermediate(HR=1.822, 95% CI: 1.529–2.135, *p <*0.01) and LIPI poor(HR=1.593, 95% CI: 1.426–1.826, *p <*0.01) were identified as independent risk factors for irAEs (**Figure 2B**). Next, we constructed predictive nomogram models for OS and irAEs based on Cox multivariate analysis (**Figures 3A, E**).

### 3.4 Model development and evaluation for OS

**Figure 4:**
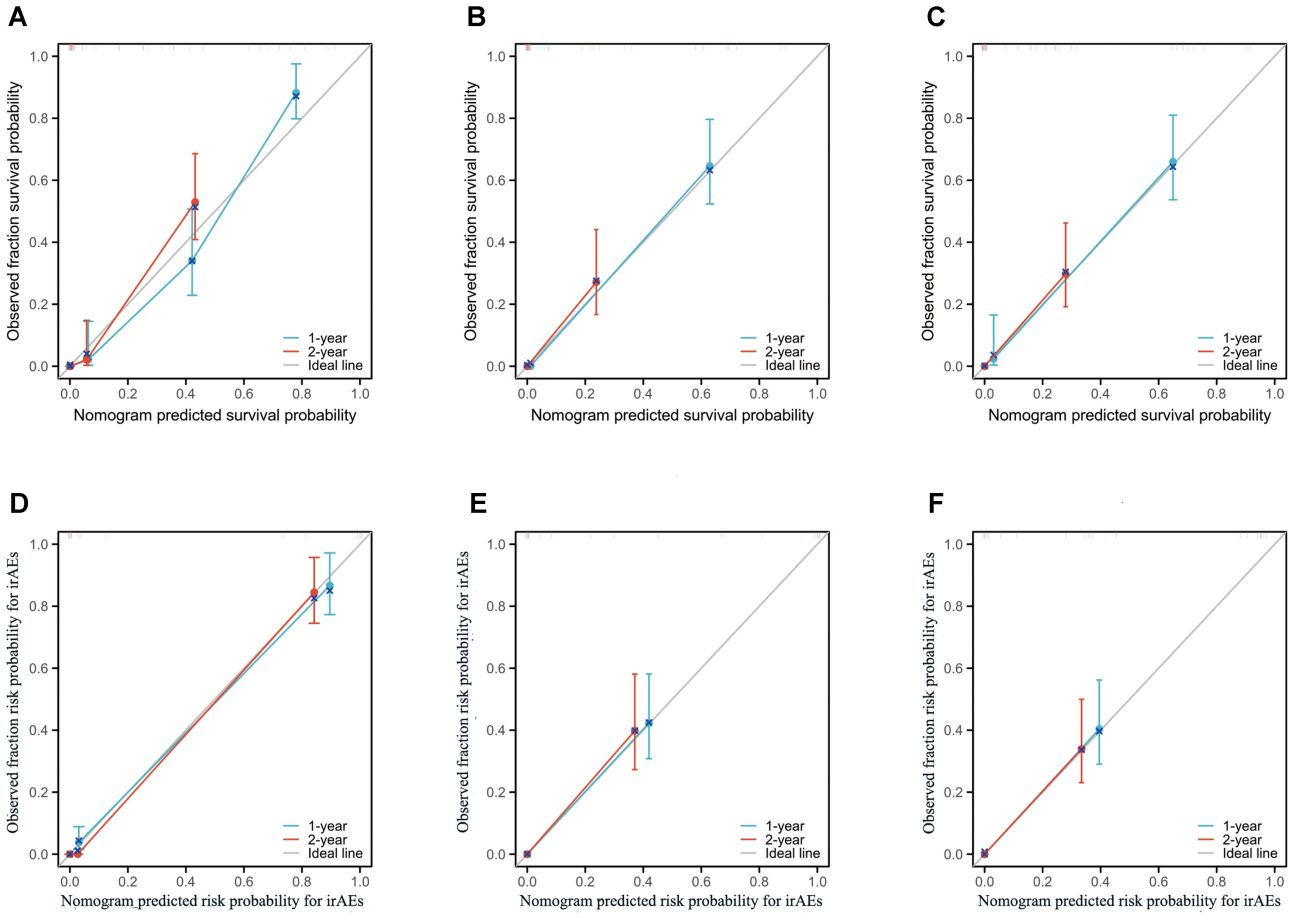
Calibration plots of the nomogram models for overall survival (OS) and immune-related adverse events (irAEs) in advanced non-small cell lung cancer (NSCLC) patients treated with Sintilimab. **(A–C)** Calibration plots for 1-year and 2-year OS predictions in the training set **(A)**, internal validation set **(B)**, and external validation set **(C)**. **(D–F)** Calibration plots for 1-year and 2-year irAEs risk predictions in the training set **(D)**, internal validation set **(E)**, and external validation set **(F)**.

**Figure 5:**
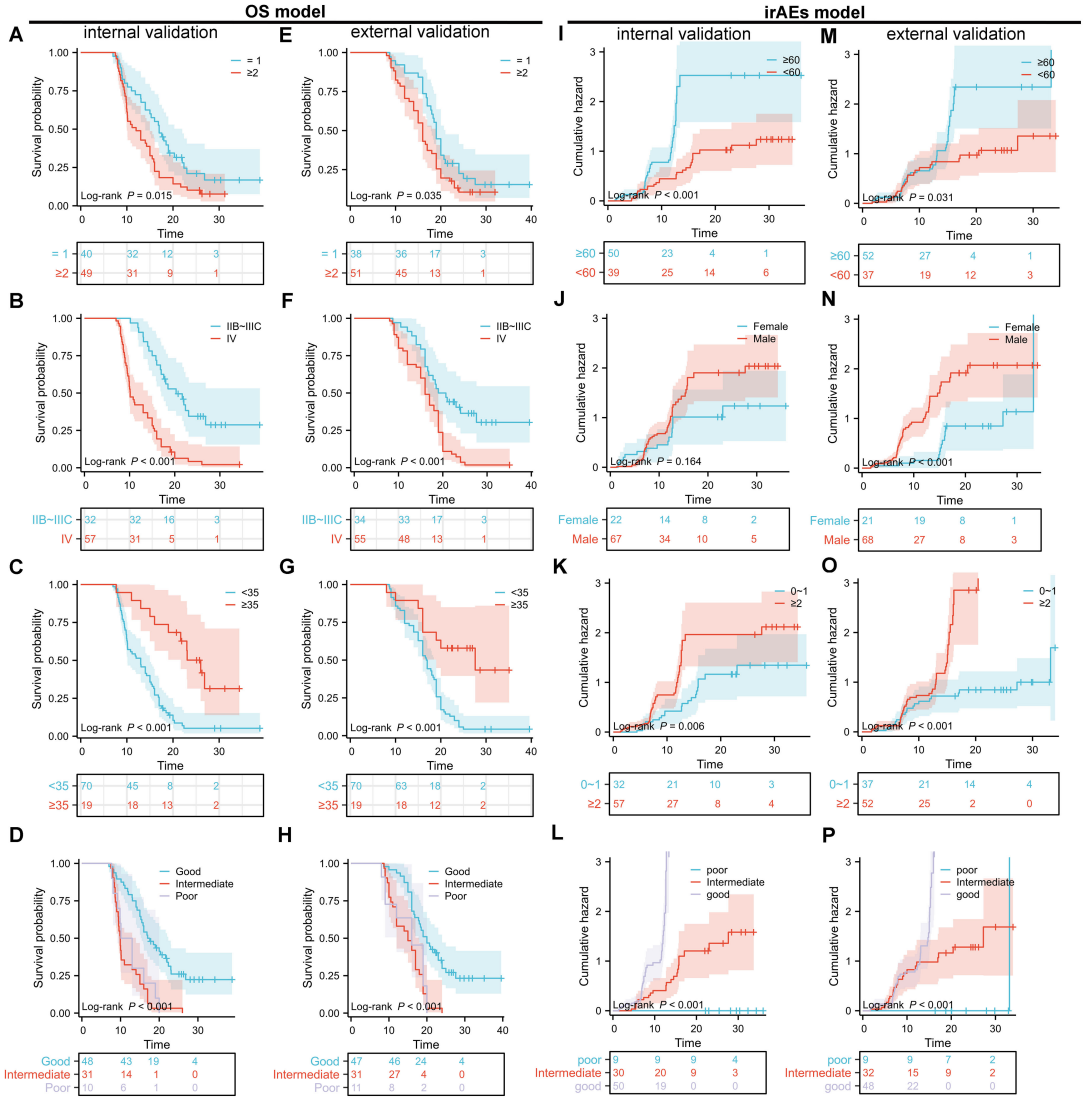
Kaplan-Meier (KM) survival curves for overall survival (OS) and cumulative incidence curves for immune-related adverse events (irAEs) in advanced non-small cell lung cancer (NSCLC) patients treated with sintilimab based on the nomogram models. **(A–D)** KM survival curves for the internal validation set of the OS nomogram model, stratified by treatment line **(A)**, clinical staging **(B)**, albumin level **(C)**, and lung immune prognostic index (LIPI) grouping **(D)**. **(E–H)** KM survival curves for the external validation set of the OS nomogram model, stratified by treatment line **(E)**, clinical staging **(F)**, albumin level **(G)**, and LIPI grouping **(H)**. **(I–P)** Cumulative incidence curves for the internal validation set of the irAEs nomogram model, stratified by age **(I)**, sex **(J)**, Eastern Cooperative Oncology Group performance status (ECOG PS) **(K)**, and LIPI grouping **(L)**. **(M–P)** Cumulative incidence curves for the external validation set of the irAEs nomogram model, stratified by age **(M)**, sex **(N)**, ECOG PS **(O)**, and LIPI grouping **(P)**.

The performance of the OS nomogram was evaluated by ROC curve analysis. The area under the curve (AUC) values for 1- and 2-year OS predictions demonstrated strong discriminatory ability: 0.826 and 0.770 in the training set (**Figure 3B**), 0.850 and 0.834 in the internal validation set (**Figure 3C**), and 0.837 and 0.805 in the external validation set (**Figure 3D**). The C-index also demonstrated excellent calibration, with values of 0.778 in the training set (**Figure 4A**), 0.793 in the internal validation set (**Figure 4B**), and 0.790 in the external validation set (**Figure 4C**). Calibration plots showed strong agreement between predicted and observed outcomes, with minimal deviation from the ideal diagonal, indicating high reliability of the model. Points above the diagonal reflected overestimation, while points below represented underestimation; the close alignment along the diagonal confirmed the model’s clinical utility. Kaplan-Meier survival analysis further validated the model’s superiority over single-factor assessments, as shown by four individual prognostic factors in the validation cohort (**Figures 5A–H**).

### 3.5 Model development and evaluation for irAEs

For the irAEs nomogram, ROC curves showed AUC values for 1- and 2-year predictions of 0.816 and 0.768 in the training set (**Figure 3F**), 0.835 and 0.822 in the internal validation set (**Figure 3G**), and 0.823 and 0.754 in the external validation set (**Figure 3H**). The C-index values were 0.805 for the training set (**Figure 4D**), 0.825 for the internal validation set (**Figure 4E**), and 0.775 for the external validation set (**Figure 4F**), demonstrating good calibration. Calibration curves showed close alignment with the ideal diagonal, confirming the model’s predictive accuracy. We analyzed irAEs occurrence as the primary endpoint (analogous to mortality in survival analysis). The incidence of irAEs was assessed, accounting for other clinical events. Kaplan-Meier estimates (**Figures 5I–P**) and cumulative incidence curves consistently demonstrated the nomogram’s superior predictive accuracy compared to individual prognostic factors in both time-to-event and risk probability assessments.

### 3.6 Clinical utility and net benefit analysis

**Figure 6:**
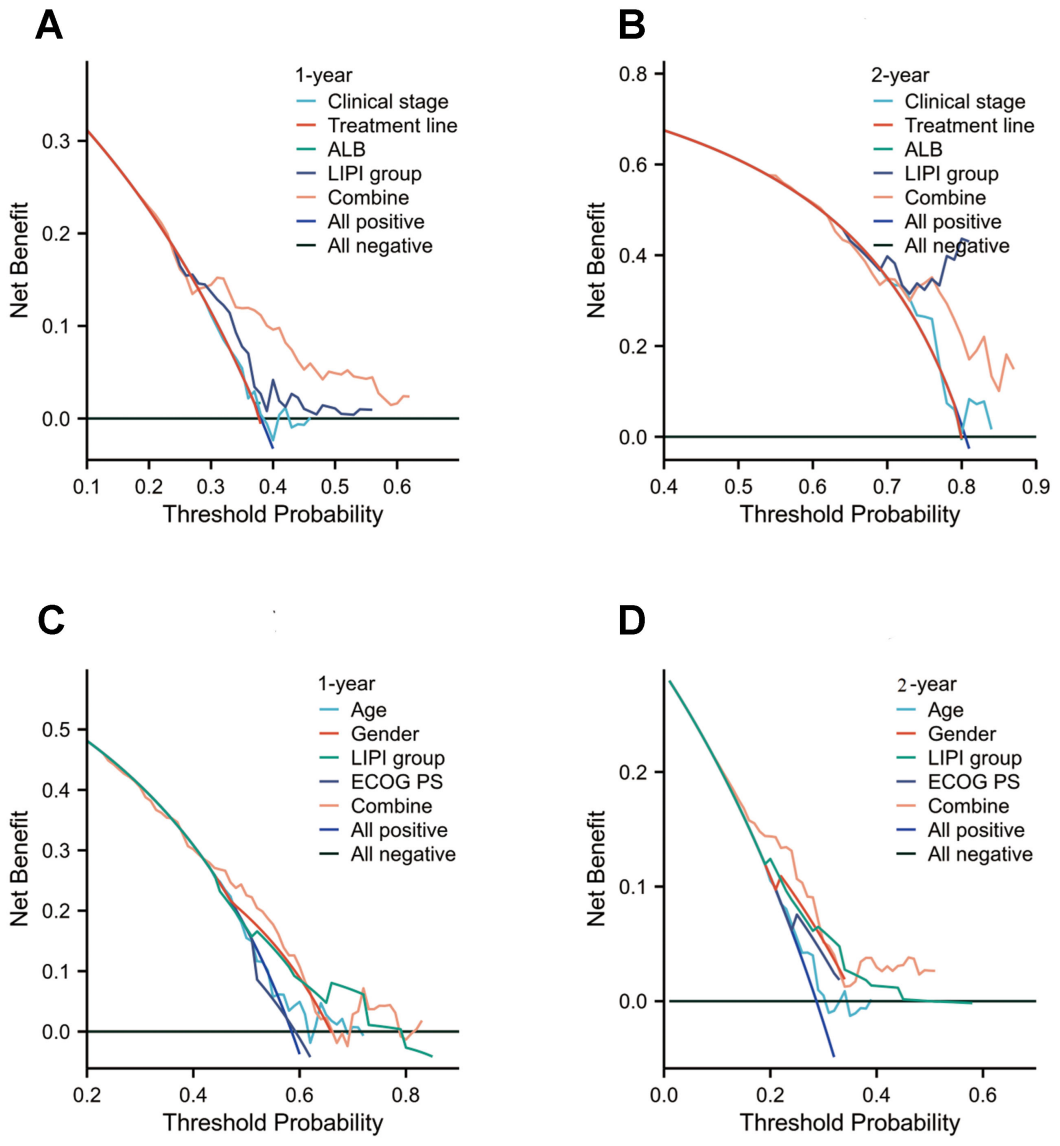
Decision curve analysis (DCA) of the nomogram models for overall survival (OS) and immune-related adverse events (irAEs) in advanced non-small cell lung cancer (NSCLC) patients treated with Sintilimab. **(A, B)** DCA for 1-year **(A)** and 2-year **(B)** OS predictions in the training set. **(C, D)** DCA for 1-year **(C)** and 2-year **(D)** irAEs risk predictions in the training set.

We assessed the clinical utility of the combined predictive model using DCA to quantify net benefit. The model demonstrated significantly higher net benefit across clinically relevant threshold probabilities compared to individual variables (**Figure 6**), confirming its superior capacity to: ① guide therapeutic decisions and ② enhance risk stratification accuracy. These findings corroborate existing evidence favoring integrated prediction models over single-variable approaches. Importantly, our analysis provides empirical validation of the added value achieved by incorporating multiple prognostic factors, offering clinicians a robust tool for high-risk patient identification.

## 4 Discussion

While sintilimab has shown significant clinical efficacy in the treatment of NSCLC, its widespread application remains limited by two primary challenges: (1) the lack of specific patient selection criteria and (2) difficulties in managing irAEs. Precise prediction of therapeutic response is therefore critical for optimizing patient stratification and minimizing the risk of irAEs. Our previous research demonstrated sintilimab’s safety and efficacy in a well-defined subgroup of NSCLC patients (20), identifying clinical stage, treatment lines, LIPI score, and ALB levels as significant predictors of OS. Additionally, age, sex, ECOG-PS, and LIPI score were independently associated with irAEs risk. Using data from the First Affiliated Hospital of Bengbu Medical University, we developed prognostic nomograms for OS and irAEs in a training cohort, which were subsequently validated internally in a separate cohort and externally using independent datasets from Hefei Eighth People’s Hospital and Suzhou Municipal Hospital. Both internal and external validation confirmed the models’ strong generalizability (21), providing a clinically actionable framework for guiding treatment decisions in sintilimab-treated NSCLC patients.

This retrospective study initially enrolled 1,197 patients with advanced or metastatic NSCLC. To address baseline differences in variables such as age, sex, ECOG-PS, LIPI grouping, clinical stage, treatment lines, and ALB levels, propensity scores were calculated for each patient based on their clinical characteristics. These scores were then used to match patients across the training, internal validation, and external validation sets using PSM (22). PSM is a robust statistical method that minimizes bias in retrospective studies, approximating the validity of randomized controlled trials (23, 24). Through rigorous study design, meticulous data processing, and appropriate statistical methods, we enhanced the reliability and generalizability of our findings. While traditional PSM typically compares two patient groups, our study required simultaneous matching across three cohorts.

While prior research has predominantly focused on either OS or irAEs in isolation (25, 26), this study innovatively integrates the LIPI to develop a dual-prediction model that concurrently estimates OS and irAEs in NSCLC patients receiving sintilimab. Our study revealed a clinically significant paradox: NSCLC patients with favorable LIPI scores experienced both significantly longer survival and a higher incidence of irAEs following sintilimab treatment compared to those with intermediate or poor LIPI scores. This presents a therapeutic dilemma—whether to prioritize survival benefits or minimize irAEs risks in patients with favorable LIPI scores. These findings align with existing literature (27) and may reflect heightened immune system activity in patients with favorable LIPI scores (28), which could lead to both enhanced immunotherapy responses and increased susceptibility to irAEs (29, 30). To address this challenge, our constructed OS and irAEs nomograms enable quantitative risk-benefit assessments, facilitating more informed therapeutic decisions. While multivariate Cox and Kaplan-Meier analyses confirmed LIPI as an independent prognostic factor, its standalone predictive accuracy remains limited (31). Comprehensive patient assessment requires integrating multiple clinical parameters, including age, sex, ECOG-PS, clinical stage, treatment lines, and ALB levels (32). This multidimensional approach improves personalized treatment optimization by providing more accurate outcome predictions and supporting balanced therapeutic decision-making.

Our study also identified sex as an independent risk factor for irAEs, with male patients exhibiting significantly higher irAEs incidence compared to females. This finding corroborates prior evidence of sex-based differences in immunotherapy response (33), potentially attributable to intrinsic immune system variations, hormonal influences, or genetic predispositions. Further research is warranted to elucidate these mechanisms and optimize personalized treatment strategies. In line with Sonehara et al. (34), our study validated the baseline LIPI score as an independent predictor of irAEs in NSCLC patients receiving PD-1 inhibitors. However, a retrospective study of elderly NSCLC patients treated with PD-1 inhibitors (35) reported no significant correlation between baseline LIPI classification and irAEs risk, though changes in LIPI scores during treatment significantly predicted irAEs occurrence. While our findings differ from these results, they highlight an important limitation of our study: we did not analyze dynamic changes in LIPI scores following sintilimab treatment (36). Future studies should incorporate serial LIPI assessments to better characterize its predictive value for irAEs risk.

Current literature demonstrates that both the Prognostic Nutritional Index (PNI) and LIPI significantly correlate with OS and progression-free survival (PFS) in advanced NSCLC (37). Notably, patients with poor LIPI scores show significantly worse outcomes independent of their PD-L1 tumor proportion score (TPS), suggesting LIPI’s superior predictive value in chemoimmunotherapy contexts. Compared with PNI, LIPI exhibits greater predictive accuracy for chemoimmunotherapy outcomes in advanced NSCLC. Its particularly strong correlation with treatment response among patients with low PD-L1 expression positions LIPI as a clinically valuable decision-making tool.

This retrospective study has inherent constraints, including potential selection/information bias and inability to establish causality due to its observational design. Data quality was inconsistent, with possible missing records. Both validation cohorts were relatively small (n=89 each), limiting statistical power and preventing subgroup analyses (e.g., LIPI model performance across irAE types/severities). The analyzed variables were insufficiently comprehensive, omitting potential predictors like TMB and cytokine levels. Larger prospective studies are needed to validate these findings.

## 5 Conclusion

This study successfully developed and validated prognostic and toxicity-prediction nomograms for OS and irAEs in NSCLC patients treated with sintilimab. These nomograms provide a valuable tool for personalized treatment planning and risk stratification. Future multicenter studies should evaluate their generalizability across diverse ethnic populations, clinical settings, and treatment regimens to further optimize their clinical application.

## Data Availability

The original contributions presented in the study are included in the article/**Supplementary Material**. Further inquiries can be directed to the corresponding authors.

## Supplementary material

Supplementary Figure 1 Kaplan-Meier (KM) survival curves for overall survival (OS) and cumulative incidence curves for immune-related adverse events (irAEs) in advanced non-small cell lung cancer (NSCLC) patients treated with sintilimab. **(A–C)** KM survival curves for the OS nomogram model, stratified by training set **(A)**, internal validation **(B)**, and external validation **(C)**. **(D–F)** Cumulative incidence curves for the irAEs nomogram model, stratified by training set **(D)**, internal validation **(E)**, and external validation **(F)**.
