# Supplementary figures and images for: Development of a lung immune prognostic index-based nomogram model for predicting overall survival and immune-related adverse events in non-small cell lung cancer patients treated with sintilimab
Source: Front Immunol. 2025 May 8;16:1569689. doi: 10.3389/fimmu.2025.1569689 (PMC12095032; doi:10.3389/fimmu.2025.1569689)

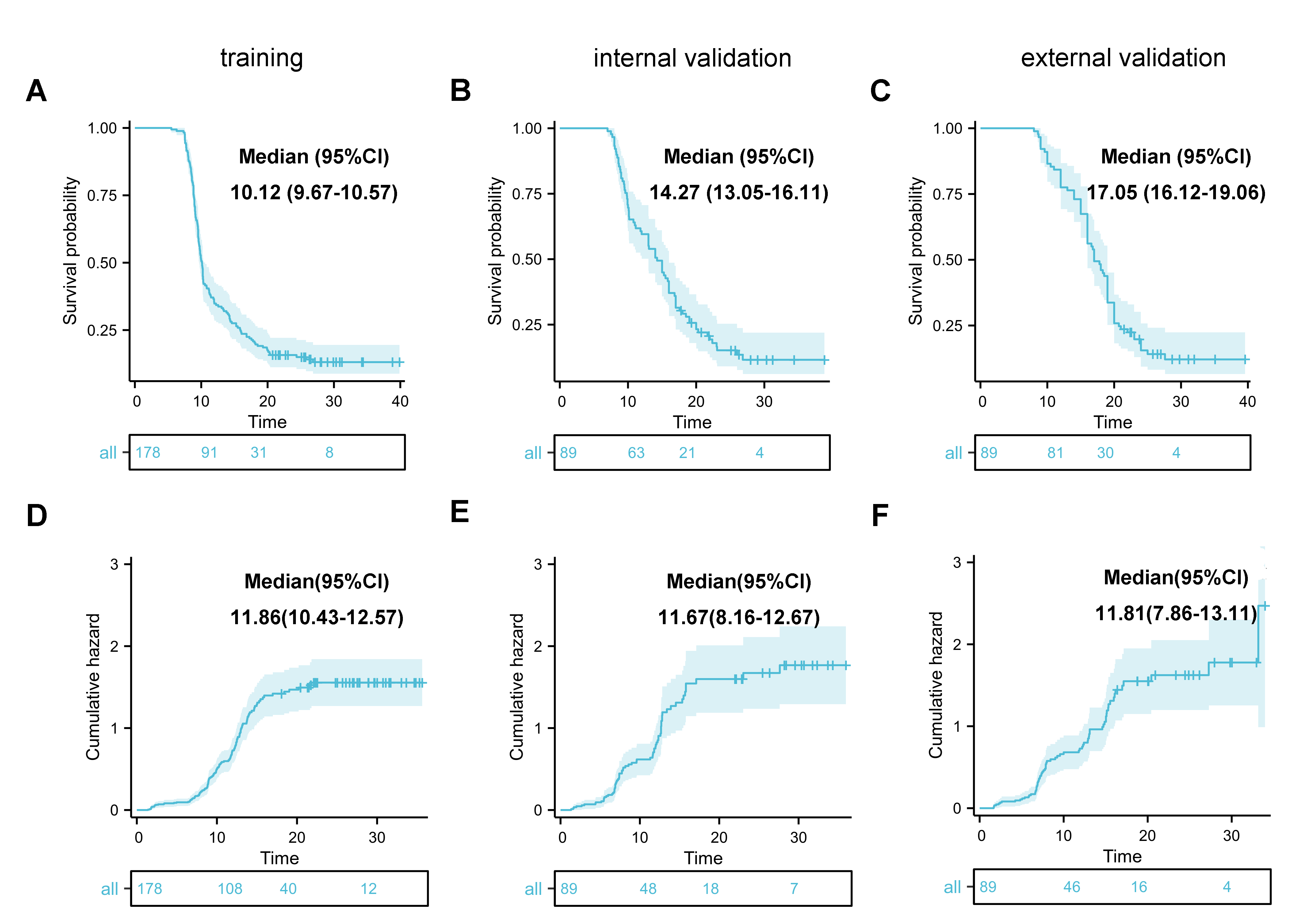

Supplement: Supplementary Figure 1 — Kaplan-Meier (KM) survival curves for overall survival (OS) and cumulative incidence curves for immune-related adverse events (irAEs) in advanced non-small cell lung cancer (NSCLC) patients treated with sintilimab. (A–C) KM survival curves for the OS nomogram model, stratified by training set (A), internal validation (B), and external validation (C). (D–F) Cumulative incidence curves for the irAEs nomogram model, stratified by training set (D), internal validation (E), and external validation (F). [file Image1.tif]
