# Supplementary material for: Development of a lung immune prognostic index-based nomogram model for predicting overall survival and immune-related adverse events in non-small cell lung cancer patients treated with sintilimab
Source: Front Immunol. 2025 May 8;16:1569689. doi: 10.3389/fimmu.2025.1569689 (PMC12095032; doi:10.3389/fimmu.2025.1569689)
Supplement: Supplementary file 2 [file Table1.docx]

**Table S1.** Identification of independent predictors of overall survival (OS) in advanced non-small cell lung cancer (NSCLC) patients treated with sintilimab.

| **Characteristics** | **HR (95% CI)** | P value |
| --- | --- | --- |
| **Age** |  |  |
| ≥ 60 | Reference |  |
| <60 | 1.058(0.824 - 1.316) | < 0.01 |
| **Sex** |  |  |
| Male | Reference |  |
| Female | 0.950 (0.647 - 1.394) | 0.351 |
| **ECOG PS** |  |  |
| 0~1 | Reference |  |
| ≥2 | 2.537 (1.637 - 3.931) | 0.682 |
| **Smoking** |  |  |
| Yes | Reference |  |
| No | 1.321 (0.937 - 2.007) | 0.091 |
| **Pathological type** |  |  |
| Squamous cell carcinoma | Reference |  |
| Non-squamous cell carcinoma | 0.859 (0.574 - 1.249) | 0.733 |
| Others | 1.105 (0.853 - 1.408) | 0.562 |
| **Clinical stage** |  |  |
| Stage IIIB~IIIC | Reference |  |
| Stage IV | 1.327 (1.026 - 1.596) | < 0.01 |
| **History of radiotherapy** |  |  |
| Yes | Reference |  |
| No | 2.016 (1.371 - 3.527) | 0.738 |
| **Treatment lines** |  |  |
| 1 | Reference |  |
| ≥2 | 1.487 (1.368 - 1.703) | < 0.01 |
| **LIPI** |  |  |
| Good | Reference |  |
| Intermediate | 1.665 (1.232 - 1.869) | < 0.01 |
| Poor | 1.506 (1.153 - 1.826) | < 0.01 |
| **Tumor stage** |  |  |
| 0-2 | Reference |  |
| 3-4 | 2.128 (1.867 - 2.426) | < 0.01 |
| **EGFR/ALK** |  |  |
| Negative | Reference |  |
| Unknown | 1.845(1.371 - 2.436) | 0.602 |
| PD-L1 TPS |  |  |
| < 1% | Reference |  |
| ≥ 1% | 0.558(0.351 - 0.862) | 0.125 |
| Unknown | 0.706(0.529 - 1.008) | 0.253 |
| **Hemoglobin** |  |  |
| <110 | Reference |  |
| ≥110 | 0.950 (0.647 - 1.394) | 0.792 |
| **Albumin** |  |  |
| <35 | Reference |  |
| ≥35 | 1.327(1.029 - 1.586) | < 0.01 |
| **CA 199** |  |  |
| <37 | Reference |  |
| ≥37 | 1.445 (0.979 - 2.133) | 0.064 |

Abbreviations: ECOG-PS, Eastern Cooperative Oncology Group performance score; LIPI, lung immune prognostic index; EGFR, Epidermal Growth Factor Receptor; ALK, Anaplastic Lymphoma Kinase; PD-L1 TPS, Programmed Death-Ligand 1 Tumor Proportion Score; CA199, carbohydrate antigen 199.
