# Supplementary material for: Development of a lung immune prognostic index-based nomogram model for predicting overall survival and immune-related adverse events in non-small cell lung cancer patients treated with sintilimab
Source: Front Immunol. 2025 May 8;16:1569689. doi: 10.3389/fimmu.2025.1569689 (PMC12095032; doi:10.3389/fimmu.2025.1569689)
Supplement: Supplementary file 3 [file Table2.docx]

Table S2. Identification of independent predictors of immune-related adverse events (irAEs) in advanced non-small cell lung cancer (NSCLC) patients treated with sintilimab.

| **Characteristics** | **HR (95% CI)** | P value |
| --- | --- | --- |
| **Age** |  |  |
| ≥ 60 | Reference |  |
| <60 | 1.688(1.236 - 2.132) | < 0.01 |
| **Sex** |  |  |
| Male | Reference |  |
| Female | 1.625 (1.321 - 2.086) | < 0.01 |
| **ECOG PS** |  |  |
| 0~1 | Reference |  |
| ≥2 | 3.358 (2.765 - 4.532) | < 0.01 |
| **Smoking** |  |  |
| Yes | Reference |  |
| No | 1.671 (1.213 - 2.426) | 0.103 |
| **Pathological type** |  |  |
| Squamous cell carcinoma | Reference |  |
| Non-squamous cell carcinoma | 0.635 (0.326 - 1.012) | 0.632 |
| Others | 0.826 (0.579 - 1.233) | 0.758 |
| **Clinical stage** |  |  |
| Stage IIIB~IIIC | Reference |  |
| Stage IV | 1.982 (1.428 - 2.392) | < 0.01 |
| **History of radiotherapy** |  |  |
| Yes | Reference |  |
| No | 3.125 (2.5723 - 4.658) | 0.851 |
| **Treatment lines** |  |  |
| 1 | Reference |  |
| ≥2 | 0.781 (0.325 - 1.005) | 0.525 |
| **LIPI** |  |  |
| Good | Reference |  |
| Intermediate | 1.957(1.657 - 2.361) | < 0.01 |
| Poor | 1.757(1.526 - 1.933) | < 0.01 |
| **Tumor stage** |  |  |
| 0-2 | Reference |  |
| 3-4 | 2.657 (2.328 - 2.863) | < 0.01 |
| **EGFR/ALK** |  |  |
| Negative | Reference |  |
| Unknown | 1.335(0.958 - 1.723) | 0.751 |
| **PD-L1 TPS** |  |  |
| < 1% | Reference |  |
| ≥ 1% | 0.651(0.367 - 0.922) | 0.276 |
| Unknown | 0.856(0.681 - 1.225) | 0.329 |
| **Hemoglobin** |  |  |
| <110 | Reference |  |
| ≥110 | 1.335 (0.926 - 1.605) | 0.382 |
| **Albumin** |  |  |
| <35 | Reference |  |
| ≥35 | 0.815 (0.639 - 1.217) | 0.581 |
| **CA 199** |  |  |
| <37 | Reference |  |
| ≥37 | 1.819 (1.325 - 2.491) | 0.098 |

Abbreviations: ECOG-PS, Eastern Cooperative Oncology Group performance score; LIPI, lung immune prognostic index; EGFR, Epidermal Growth Factor Receptor; ALK, Anaplastic Lymphoma Kinase; PD-L1 TPS, Programmed Death-Ligand 1 Tumor Proportion Score; CA199, carbohydrate antigen 199.
